# Interleukin-6 and Interleukin-15 as Possible Biomarkers of the Risk of Autoimmune Diabetes Development

**DOI:** 10.1155/2019/4734063

**Published:** 2019-10-20

**Authors:** Katarzyna Siewko, Rafal Maciulewski, Anna Zielinska-Maciulewska, Anna Poplawska-Kita, Piotr Szumowski, Natalia Wawrusiewicz-Kurylonek, Danuta Lipinska, Robert Milewski, Maria Gorska, Adam Kretowski, Malgorzata Szelachowska

**Affiliations:** ^1^Department of Endocrinology, Diabetology and Internal Medicine, Medical University of Białystok, Białystok, Poland; ^2^Department of Nuclear Medicine, Medical University of Białystok, Białystok, Poland; ^3^Department of Statistics and Medical Informatics, Medical University of Białystok, Białystok, Poland

## Abstract

**Aim:**

The aim of our study was to assay circulating interleukin-15 (IL-15) and interleukin-6 (IL-6) levels and insulin resistance measured by two different methods in newly diagnosed autoimmune diabetes (AD) patients, their I° relatives, and healthy controls.

**Material and Methods:**

The group studied consisted of 54 patients with AD (28 with Latent Autoimmune Diabetes in Adults (LADA) and 26 with type 1 diabetes (T1D)), 70 first-degree relatives, and 60 controls. IL-6, IL-15, and anti-islet antibodies concentrations were measured by ELISA method. Homeostatic model assessment-insulin resistance (HOMAIR) and estimated glucose disposal rate (eGDR) were calculated.

**Results:**

The patients with AD had significantly higher IL-15, IL-6, and HOMAIR and lower eGDR than the controls (*p* < 0.001, respectively) and first-degree relatives (*p* < 0.001, respectively). Significantly higher IL-15 and IL-6 were shown in the relatives with positive Ab as compared to the relatives without antibodies (*p* < 0.001, respectively) and the controls (*p* < 0.001, respectively). IL-15 negatively correlated with eGDR (*r* = −0.436, *p* = 0.021) in LADA and positively with HOMAIR in LADA and T1D (*r* = 0.507, *p* < 0.001; *r* = 0.4209, *p* < 0.001).

**Conclusions:**

Significantly higher IL-15 and IL-6 concentrations, HOMAIR, and markedly lower eGDR in newly diagnosed AD patients and first-degree relatives with positive anti-islet antibodies might suggest the role of these pro-inflammatory cytokines and insulin resistance in the pathogenesis of autoimmune diabetes. IL-15 and IL-6 might be used as biomarkers of the risk of autoimmune diabetes development, in particular IL-15 for LADA. Both methods of IR measurement appear equally useful for calculating insulin resistance in autoimmune diabetes.

## 1. Background

The pathogenesis of autoimmune diabetes is complex and includes both genetic predisposition and environmental factors, such as viral infections, diet, and toxins [1]. After an autoimmune attack by anti-islet antibodies, the pancreas is infiltrated by immune cells, such as CD4+ and CD8+ lymphocytes, as well as antigen-presenting cells—macrophages, and dendritic cells [2, 3]. Elevated circulating levels of pro-inflammatory peptides produced by mononuclear cells, observed in patients with AD, strongly suggest a role of a disturbed balance between pro-inflammatory cytokines, such as interleukin-1 (IL-1), interleukin-2 (IL-2), tumor necrosis factor alpha (TNF-*α*), and interferon-*γ* (IFN-*γ*), and those with anti-inflammatory properties, such as interleukin-4 (IL-4), interleukin-10 (IL-10), and tumor growth factor-*β* (TGF-*β*) in the pathogenesis of both types of autoimmune diabetes [4–6]. However, the mechanisms involved in the attraction of mononuclear cells to the islets and in the destruction of pancreatic beta cells have not been clear to date. Some authors described the role of insulin resistance in the pathogenesis of T1D [4, 7–9]. Increased prevalence of abdominal obesity and the metabolic syndrome, observed in patients with type 1 diabetes, suggests an association between insulin resistance and the risk of chronic diabetic complications. It is generally known that obesity is associated with increased secretion of mediators that reduce insulin-mediated glucose uptake and insulin sensitivity, such as TNF-*α*, IFN-*γ*, and IL-1*β*. However, the relationship between obesity, low-grade inflammation, and the onset of autoimmune diabetes has not been well characterized [4–6].

Interleukin-6 and interleukin-15 are cytokines produced by monocytes/macrophages during innate and adaptive immune responses. Both interleukins play an important role in the pathogenesis of several chronic autoimmune diseases: psoriasis, multiple sclerosis, rheumatoid arthritis, and ulcerative colitis. However, there is no direct evidence that IL-6 and IL-15 are involved in the diabetogenic process. IL-15 induces proliferation of CD4+ and CD8+ T cells and secretion of INF-*γ* and TNF-*α* by natural killer (NK) cells and increases immunoglobulin secretion [7–9].

Rothe et al. observed that in nonobese diabetic (NOD) mice, insulitis was associated with up-regulation of IL-15 gene expression [10]. Increased concentration of IL-15 was also found in diabetic mice by Gupta et al. [11]. Conversely, some studies have shown that IL-15 treatment reduces the development of diabetes in NOD mice, probably via a stimulatory effect of IL-15 on NK cells, suggesting a protective role for this cytokine in type 1 diabetes [12].

IL-6 regulates glucose homeostasis by stimulating bowel L cells and pancreatic *α* cells to produce and secrete glucagon-like peptide-1 (GLP-1), thereby improving insulin secretion [12, 13]. Some authors demonstrated an increased IL-6 concentration in T1D, while others found no differences in IL-6 concentrations between T1D patients and healthy controls, or even lower IL-6 concentration in T1D [14]. In this light, the role of both cytokines in the pathogenesis of autoimmune diabetes seems still discursive. 

Insulin resistance is increasingly more often ascribed a role in the pathogenesis of autoimmune diabetes. The main mechanism proposed is the inhibition of insulin signaling, leading to increased inflammatory processes, amino acids, and free fatty acids concentrations. Latest decades yielded number of hypotheses about the interrelationship between insulin resistance and development of type 1 diabetes such as “Fertile Field,” “accelerator,” and double diabetes hypothesis. But until now, the precise role of IR in development and progression of type 1 diabetes has not been completely understood [15–17]. The euglycemic-hyperinsulinemic clamp as a reference method for measurement of insulin sensitivity is used, but because of invasive procedure is not in practical for use. The most frequently used formula is homeostatic model assessment-insulin resistance (HOMAIR). However, this index is not usually applicable in autoimmune diabetic patients because of their use of exogenous insulin. In 2000, Williams et al. described a method of measuring insulin resistance in T1D. On the basis of prior studies, they used some components, such as hypertension, waist-to-hip ratio (WHR), and glycated hemoglobin (HbA1c), to calculate a formula of estimated glucose disposal rate (eGDR). Further studies showed that eGDR results were similar with results of euglycemic-hyperinsulinemic clamp [17].

The aim of our study was to assay circulating IL-15 and IL-6 levels in patients with newly diagnosed AD, their first-degree relatives, and healthy controls in comparison with the presence of anti-islet antibodies and insulin resistance. All persons chosen for the study were patients with newly diagnosed autoimmune diabetes without hypoglycemic treatment, so we attempted to compare two insulin resistance indices such as HOMAIR and eGDR and try to verify these methods with regard to sensitivity and accuracy.

## 2. Methods

The group studied consisted of 54 persons in the median age of 34.5 years (18–55 years) and with median BMI of 22.5 kg/m^2^ (17–36 kg/m^2^) with newly diagnosed autoimmune diabetes. Diabetes was diagnosed based on incidental glucose >200 mg/dl and accompanying symptoms, for example, ketoacidosis (according to criteria WHO 1999).

On the basis of the patient's age, the presence of glutamic acid decarboxylase antibodies (GADA), insulin antibodies (IAA), and/or tyrosine phosphatase antibodies (IA-2A), and the result of glucagon stimulation test, 28 of them were classified as having latent autoimmune diabetes in adults (LADA) and 26 as T1D. All AD patients had positive titers for at least one type of autoantibodies against beta cells. The LADA subgroup was selected on the basis of age, over 35, low-to-normal initial C-peptide levels, and no doubling in 6 minutes of glucagon test. LADA patients also responded to noninsulin glucose-lowering agents as the first line of antidiabetic treatment. Meanwhile, the patients with type 1 diabetes were younger (up to 34 years old), had the rapid onset disease (higher glucose concentration with ketoacidosis), and had lower initial C-peptide levels with no stimulation in the glucagon test in comparison with LADA patients.

On the study were qualified only persons with newly diagnosed autoimmune diabetes before initiation of any hypoglycemic treatment. In patients, especially with type 1 diabetes, with high blood glucose levels (>250 mg/dl) blood sampling and glucagon-stimulating tests were performed on fasting after prior metabolic adjustment (glycemic control and fluid and electrolyte management). Patients with diabetes secondary to another condition (e.g., steroid use, Cushing's disease, acute and chronic pancreatitis), liver cirrhosis, advanced renal failure, cancers, advanced heart failure (NYHA III–IV), or acute inflammation (based on CRP indication) were excluded from this study.

Seventy first-degree relatives of patients with AD in the median age of 33.0 years (18–60 years) and with median BMI of 21.9 kg/m^2^ (17 –37.5 kg/m^2^), as well as 60 healthy controls (median age—38.5 years [18–60 years]) and median BMI—22.7 kg/m^2^ [18–36.3 kg/m^2^], with no history of diabetes and other autoimmune disorders, were also recruited. All relatives and controls underwent a 75 g oral glucose tolerance test (OGTT), and the persons with abnormal results were excluded. An informed consent was obtained from all participants, and the study protocol was approved by the local ethics board (Ethics Committee of the Medical University of Bialystok).

Serum anti-islet antibodies (GADA, IAA, IA-2A) concentrations were measured by ELISA method (EUROIMMUN, Poland, test sensitivity—95%, specificity—97%). Cutoff values for positive antibody values were calculated as the 99^th^ percentile of each antibody level in 350 nondiabetic persons, and they were as follows: 1.0 U/ml for GADA, 9.8 U/ml for IAA, 0.75 U/ml for IA-2A. IL-15 and IL-6 were measured by ELISA method (R and D, USA).

Insulin resistance was calculated using the estimated glucose disposal rate (eGDR), according to the equation: eGDR = 24.31 − (12.22 × waist-to-hip ratio) – (3.29 × HT) – (0.57 × HbA1c) in mg/kg^−1^/min ^−1^, where WHR (waist-to-hip ratio), HbA1c (glycated hemoglobin), and HT (history of hypertension).

Hypertension is defined as blood pressure ≥140/90 mmHg or as previously treated hypertension. Glycated hemoglobin level (HbA1c) was measured using high-performance liquid chromatography (HPLC) (BIO-RAD Laboratories, Germany). Lower eGDR indicates greater insulin resistance.

Fasting plasma glucose concentrations using glucose oxidase method (CORMAY, Poland) and fasting insulin concentrations using ELISA method (Bio Source Europe) were measured, and HOMAIR (Homeostatic Model Assessment of Insulin Resistance) index was calculated according to the formula: fasting insulinemia (mU/ml) × fasting plasma glucose (mmol/l)/22.5. HOMAIR above 2 testifies to insulin resistance.

## 3. Statistical Analysis

Statistical analysis was performed with the use of STATISTICA 10.0 software (StatSoft, Tulsa, USA). Before the analysis, data were tested for normality of distribution with the Shapiro–Wilk test. Kruskal–Wallis test was applied to compare differences between groups and Spearman correlations to relationships between variables. *P* value lower than 0.05 was considered to be statistically significant.

## 4. Results

The patients with autoimmune diabetes had significantly higher concentrations of IL-15, IL-6, HOMAIR index, and markedly lower eGDR values in comparison with the controls (*p* < 0.001, respectively) (Table 1). Similar results were found between the AD group as compared to the first-degree relatives (Table 1). When the AD group was divided into the T1D and LADA subgroups, we observed significantly higher IL-15 and IL-6 concentrations (*p* < 0.001) and significantly lower IA-2A levels (*p* = 0.01) in the patients with LADA as compared to the T1D group (Table 2).

Increased concentration of at least one antibody against pancreatic islet antigens was detected in 31 (44.3%) of healthy first-degree relatives of patients with AD: IAA—in 21 relatives (23.3%); GADA—in 15 relatives (16.7%), and IA-2A—in 2 relatives (2.2%). Two antibodies (IAA and GADA) were found in 5 relatives. The group of relatives had significantly higher concentrations of GADA (*p* = 0.01), IAA (*p* < 0.001), IL-15 (*p* < 0.001) and IL-6 (*p* < 0.001), mean HOMAIR index (*p* < 0.001) as well as lower value of eGDR (*p* < 0.001) in comparison with the controls. Analyzing the subgroup of relatives with and without anti-islet antibodies, we observed significantly higher concentrations of IL-15, IL-6, and HOMAIR in the subgroup of relatives with positive anti-islet antibodies as compared to the relatives without antibodies (*p* < 0.001, *p* < 0.001, *p* = 0.02, respectively) and to the healthy controls (*p* < 0.001, respectively). eGDR values did not differ between the subgroups of relatives (Table 3). We also found significantly higher concentrations of IL-15 and markedly lower eGDR values in the patients with LADA in comparison with the relatives with positive anti-islet antibodies (*p* < 0.001, respectively). The values of eGDR were also lower in the patients with T1D than in the relatives with positive autoantibodies (*p* < 0.001). IL-6 concentrations and HOMAIR did not differ between the LADA and T1D groups and the relatives with positive antibodies. The group of relatives without antibodies had significantly higher eGDR as compared to LADA and T1D groups (*p* < 0.001, respectively).

We also observed negative correlations between eGDR and BMI in the total group with AD (*r* = −0.516; *p* < 0.001), the subgroup of LADA and T1D (*r* = −0.649, *p* = 0.001; *r* = −0.464, *p* = 0.021, respectively), first-degree relatives (*r* = −0.400, *p* < 0.001), and the controls (*r* = −0.497, *p* < 0.001). Only in the patients with LADA, serum IL-15 concentrations negatively correlated with eGDR (*r* = −0.436, *p* = 0.021). HOMAIR positively correlated with IL-15 in the LADA and T1D group (*r* = 0.507, *p* < 0.001 and *r* = 0.421, *p* < 0.001).

## 5. Discussion

The potential role of IL-15 and IL-6 in the pathogenesis of diabetes is still discursive, and only a handful of reports concerning their role in LADA are available [18–20]. Furthermore, most of these data were based on animal models. One study conducted on NOD mice showed that IL-15 reduces the cumulative incidence of diabetes, suggesting that this reduction could be due to a down-regulation of beta-cell apoptosis [18]. Other authors indicated that obese individuals have significantly lower IL-15 concentrations in comparison with normal-weight persons and that the treatment with IL-15 induced weight loss in an animal model. It is well known that weight loss reduces insulin resistance and improves glucose homeostasis, whereas IL-15 might improve glucose regulation in hyperglycemic obese animals [18–20]. It has been demonstrated that IL-15 affects NK and T cells development, function, and survival.

However, the role of NK cell in AD seems controversial. Some authors suggested that NK cells play a protective role against the development of autoimmune diabetes, whereas other data showed that their depletion can delay the onset of diabetes [21]. On the other hand, some authors highlighted the importance of pro-inflammatory cytokines in the pathogenesis of AD [22, 23]. Cardazo et al. observed that IL-1*β *and IFN-*γ* increased the expression of IL-15 mRNA in human and rat beta cells [30]. The role of IL-15 in the development of autoimmune process was also shown in NOD mice [10, 30, 31]. Our finding of elevated IL-15 concentration in newly diagnosed AD patients suggests the role of this cytokine in the pathogenesis of autoimmune diabetes in humans. Although a cross-sectional design of our study limits any speculations about potential mechanisms linking IL-15 with AD, our results also indicate that healthy first-degree relatives of patients with autoimmune diabetes had significantly higher IL-15 concentrations as compared with the controls. Consequently, taking all of the above into account, our results suggest that IL-15 could be a potential prognostic marker of autoimmune process in humans, in particular in these with LADA.

The potential role of IL-6 in the pathogenesis of autoimmune diabetes in humans is also unclear. Recent studies suggest an association between IL-6 and the development of type 1 diabetes [4, 32]. However, its concentration was correlated with abdominal obesity in humans [33]. Both Alexandraki et al. and Schloot et al. observed increased IL-6 secretion in type 1 diabetes [34, 35], whereas Ryden et al. did not find elevated concentrations of IL-6 in children with type 1 diabetes [36]. In the present study, IL-6 concentrations were significantly higher in the patients with newly diagnosed AD and their healthy first-degree relatives as compared to the healthy controls, which might support a role of this pro-inflammatory cytokine in the pathogenesis of autoimmune diabetes in humans.

The higher interleukin-15 and interleukin-6 concentrations in the group of newly diagnosed diabetes in our study could be associated with poor metabolic compensation at the moment of diagnosis, but similar results were also found in the group of first-degree relatives who did not have metabolic disturbances. It suggests that elevated levels of these cytokines are not directly connected with hyperglycemia.

It is well known that obesity is strongly associated with insulin resistance (IR) and chronic low-grade inflammatory process [37]. Clinical studies confirmed a positive correlation between BMI, the indices of IR, and circulating inflammatory mediators in humans [38–40]. Moreover, according to the accelerator hypothesis, insulin resistance due to obesity could explain an increased incidence of type 1 diabetes [19, 20]. Some authors also documented the presence of insulin resistance in type 1 diabetes and in prediabetic state [4, 21]. Our findings seem to be in line with these observations. An estimated glucose disposal rate was markedly lower as well as HOMAIR was significantly higher in the patients with newly diagnosed AD and first-degree relatives, suggesting an increase in IR in these groups. Our findings are also consistent with the results of the Diabetes Prevention Trial-Type 1 Study (DPT1) and the Childhood Diabetes in Finland Study (DiME) [41, 42]. In our observation, all study subjects had mean body mass index in the normal range and we did not find differences in BMI between group of newly diagnosed autoimmune diabetes, their first-degree relatives, and healthy controls. However, some authors suggest racial/ethnic differences in insulin resistance measured by eGDR. Relatively higher IR was found in nondiabetic African Americans and Hispanic adults than in nondiabetic nonHispanic white adults [43, 44]. All subjects in this study were nonHispanic white adults, so it confirms the above-cited observation. Other authors observed that lower eGDR in patients with T1D was associated with obesity and family history of type 2 diabetes [45]. In our study, eGDR was also negatively correlated with BMI, but there is no difference in mean body weight between the groups with newly diagnosed autoimmune diabetes, their first-degree relatives, and healthy controls. However, none of the subjects had family history of type 2 diabetes. We should also mention that standard methods used for the measurement of IR in nondiabetic subjects and patients with type 2 diabetes, such as homeostasis model assessment (HOMA), are not routinely recommended in patients with type 1 diabetes because of hypoinsulinemia [41]. Nevertheless, some authors used HOMA for calculating insulin resistance in patients with type 1 and LADA diabetes. Szepietowska et al. found lower IR in LADA compared to type 2 diabetes. Fatrima et al. recorded insulin resistance in 37.3% of persons with T1D tested using HOMA tests.

Consequently, we tried to confront both methods of insulin resistance measurement. Our observation showed that HOMAIR and eGDR results are similar. Certainly, we should take into account that exogenous insulin administration could disturb results of HOMA in patients with T1D or LADA. For this study, we recruited subjects with newly diagnosed AD before any hypoglycemic treatment, and our results demonstrated that in these patients with autoimmune diabetes, HOMA can be used as a method of IR measurement.

First-degree relatives of patients with autoimmune diabetes are the group with the highest risk of developing diabetes in the future. It is known that the preclinical period is initiated long before AD diagnosis and is characterized by the presence of autoantibodies against beta cell antigens, such as GADA, IAA, IA-2A, and zinc transporter 8 (ZnT8), as well as a decrease in the first phase of insulin secretion [43].

However, screening tests for these risk factors are not commonly used in daily clinical practice. It is also widely known that the presence of anti-islet antibodies alone is not sufficient to induce the destruction of beta cells. In the prediabetic stage, there is an activation of self-reactive lymphocytes that contribute to cell-mediated immunity by the production of pro-inflammatory cytokines [44]. In our study, the presence of at least one anti-islet antibody was shown in 31% of first-degree relatives and the most commonly found antibodies were IAA. It should be mentioned that insulin autoantibodies are usually the first antibodies detected in the relatives of diabetic patients, in particular in children [41, 42]. We also observed that the relatives with positive anti-islet antibodies had significantly higher IL-15 and IL-6 concentrations and significantly lower eGDR in comparison with the relatives without autoantibodies, which strongly suggests that this subgroup is at the highest risk of developing diabetes in the future.

## 6. Conclusions

In conclusion, our results demonstrated significantly higher concentrations of IL-15, IL-6, and HOMAIR index as well as markedly lower eGDR values in the patients with newly diagnosed AD, which may suggest the role of these pro-inflammatory cytokines and insulin resistance independent of weight in the pathogenesis of type 1 diabetes and LADA. Moreover, elevated concentrations of IL-15 and IL-6, lower eGDR value, and higher HOMAIR index found in the first-degree relatives with positive anti-islet antibodies suggest that serum IL-15 and IL-6 levels might be used as a biomarkers of AD risk and that insulin resistance could also play a role in the prediabetic stage. A positive correlation between IL-15 and HOMAIR and negative correlation with eGDR suggest that IL-15 also potentially affects insulin resistance. Our results imply also that both methods of insulin resistance measurement—HOMAIR and eGDR—emerge to be equally useful to calculate insulin resistance in autoimmune diabetes. This conclusion should to be confirmed on the larger population of autoimmune diabetic patients.

## Figures and Tables

**Table 1:** Clinical and biochemical characteristics of the group studied.

	Autoimmune diabetes (AD)	First-degree relatives (FDR)	Control group (CG)	*p* Value AD vs CG	*p* Value AD vs FDR	*p* Value FDR vs CG
N	54	70	60			
Age (years)	34.5 (18–55)	33.0 (18–60)	38.5 (18–60)	0.2	0.7	0.6
BMI (kg/m^2^)	22.5 (17–36)	21.9 (17–37.5)	22.7 (18–36.3)	0.7	0.3	0.7
GADA (U/ml)	111.8 (20.2–1879.7)	0.8 (0.6–218.7)	0.7 (0.5–0.9)	<0.001	<0.001	0.01
IAA (U/ml)	3.6 (1.3–9.3)	3.1 (4.4–17.2)	0.2 (0.1–3.6)	<0.001	0.3	<0.001
IA-2A (U/ml)	6.5 (4.8–4000)	0.6 (0.6–0.9)	0.6 (0.4–0.7)	<0.001	<0.001	0.3
eGDR (mg/kg/min)	6.5 (−0.7–11.7)	11.4 (7.0–13.9)	12.4 (6.7–13.4)	<0.001	<0.001	0.004
HOMAIR	2.4 (0.3–17.5)	1.04 (0.3–2.9)	0.7 (0.5–1.9)	<0.001	<0.001	0.02
IL-15 (pg/ml)	1.7 (0.2–7.0)	0.4 (0.2–9.1)	0.2 (0.2–3.6)	<0.001	0.02	0.02
IL-6 (pg/ml)	1.0 (0.3–9.4)	0.7 (0.2–3.3)	0.1 (0.01–4.7)	<0.001	0.01	0.6

Data are shown as medians (interquartile range).

**Table 2:** Clinical and biochemical characteristics of the subgroups with T1D and LADA.

	Type 1 diabetes (T1D)	LADA	*p* Value
N	26	28	
Age (years)	25.0 (18–34)	39 (35–55)	<0.001
BMI (kg/m^2^)	22.1 (17–33.2)	23.5 (16.9–36)	0.3
GADA (U/ml)	109.4 (20–3971.1)	139 (20.2–18792)	0.4
IAA (U/ml)	2.7 (1.3–7.6)	2.6 (1.3–9.3)	0.3
IA-2A (U/ml)	14.1 (4.9–4000)	5.6 (4.8–4000)	0.01
eGDR (mg/kg/min)	6.7 (2.6–11.7)	6.2 (−0.7–10.28)	1.00
HOMAIR	2.9 (0.5–7.3)	3.2 (0.5–8.5)	0.2
IL-15 (pg/ml)	1.5 (0.2–4.0)	3.8 (1.7–7.0)	<0.001
IL-6 (pg/ml)	25.0 (18–34)	39 (35–55)	<0.001

Data are shown as medians (interquartile range).

**Table 3:** Clinical and biochemical characteristics of the first-degree relatives' subgroups.

	Ab (+) first-degree relatives (FDR)	Ab (−) first-degree relatives (FDR)	*p* value
N	31	39	
Age (years)	32.0 (18–60)	31.0 (18–60)	0.4
BMI (kg/m^2^)	22.4 (17.0–37.5)	21.9 (18–36.4)	0.2
GADA (U/ml)	0.9 (0.7–218.7)	0.7 (0.6–0.9)	0.01
IAA (U/ml)	8.2 (4.4–13.2)	6.7 (0.8–8.7)	<0.001
IA-2A (U/ml)	0.6 (0.6–0.9)	0.6 (0.6–0.7)	0.6
eGDR (mg/kg/min)	11.5 (7.0–13.9)	11.4 (7.0–12.8)	1.0
HOMAIR	3.9 (0.5–7.3)	2.1 (0.2–8.8)	0.02
IL-15 (pg/ml)	1.7 (0.8–7.2)	0.2 (0.2–0.6)	<0.001
IL-6 (pg/ml)	1.4 (0.3–4.6)	0.3 (0.1–1.3)	<0.001

Data are shown as medians (interquartile range).

## Data Availability

All participants were recruited from the outpatient clinic or from the Department of Endocrinology, Diabetology and Internal Medicine during hospitalization. The data used to support the findings of this study are available from the corresponding author upon request.
